# Consumptive Coagulopathy in Angiosarcoma: A Recurrent Phenomenon?

**DOI:** 10.1155/2014/617102

**Published:** 2014-02-13

**Authors:** Mohamad Farid, Linda Ahn, Andrew Brohl, Angela Cioffi, Robert G. Maki

**Affiliations:** Mount Sinai Medical Center, One Gustave L. Levy Place, P.O. Box 1128, New York, NY 10029-6574, USA

## Abstract

*Objectives*. To report the prevalence of consumptive coagulopathy in angiosarcoma patients seen at a single center. *Methods*. We retrospectively reviewed case records of 42 patients diagnosed with angiosarcoma at Mount Sinai Hospital between 2000 and 2013. *Results*. Seven patients (17%) met clinical criteria for disseminated intravascular coagulation (DIC) in absence of concomitant clinical states known to cause coagulopathy or myelosuppression. In all patients who received systemic antineoplastic therapy with resultant disease response or stability, DIC resolved in tandem with clinical improvement. DIC recurred at time of disease progression in all cases. Two patients had bulky disease, defined as diameter of largest single or contiguous tumor mass measuring 5 cm or more. All patients demonstrated an aggressive clinical course with short duration of disease control and demise within 1 year. In contrast, evaluation over the same period of 17 epithelioid hemangioendothelioma patients serving as a clinical control group revealed no evidence of DIC. *Conclusion*. Angiosarcomas can be associated with a consumptive coagulopathy arising in tandem with disease activity. Vigilance for this complication will be needed in the course of often aggressive multimodality therapy. The potential utility of coagulopathy as a prognostic biomarker will need to be explored in future studies.

## 1. Introduction

Hypercoagulability is a well-characterized association of malignancy which can manifest in one of several clinical syndromes, including disseminated intravascular coagulation (DIC). DIC is characterized by aberrant activation of the coagulation cascade leading to initial hypercoagulability, then progressing to a secondary hypocoagulable state resulting from consumptive coagulopathy and secondary fibrinolysis. In patients with cancer, DIC can be proximately related to altered levels of various mediators of hemostasis (procoagulants, cytokines, and fibrinolytic mediators), in addition to tumor-related endothelial cell alterations and systemic depressions in hemostatic defenses [1]. DIC is known to complicate up to 15% of leukemias [2] and up to 7% of solid tumors, with data suggesting an association with adverse cancer prognosis [3].

Sarcomas comprise 1% of adult malignancies, and can take the form of more than 70 different histologic subtypes, each describing unique natural histories and clinical behavior [4]. The prevalence of coagulation abnormalities across the different sarcoma histotypes has not been well characterized. In a large series of more than 1000 patients with solid tumors, only 41 patients had sarcoma, of whom 2 were assessed to have DIC [3].

Angiosarcomas are aggressive malignancies of vascular origin that comprise 2–5% of sarcomas. They arise in virtually any anatomic site, including skin, breast, bone, extremities, deep soft tissues, and viscera [5]. Angiosarcomas are associated with a variety of environmental carcinogenic factors, including dyes, chronic lymphedema, and radiation, but most commonly arise sporadically. In advanced disease, effective therapies are limited and prognosis is dismal, with median survival of less than 1 year [5].

Epithelioid hemangioendotheliomas represent another group of vascular sarcomas derived from endothelial cells. Affecting less that 1 person per million of the population, these tumors most often involve the liver, usually multifocally. Distinct from angiosarcoma, they can be associated with an indolent clinical course and prolonged disease control, even in the absence of any therapy in some cases [6].

There are sporadic case reports describing the coexistence of a consumptive coagulopathy with angiosarcoma [7–11]. These have been characterized as variants of Kasabach-Merritt Phenomenon (KMP), the eponymous syndrome first described in 1941 in an infant with a giant capillary hemangioma, and henceforth sometimes loosely defined as a thrombocytopenic consumptive coagulopathy associated with an enlarging vascular neoplasm or malformation. The diagnosis of KMP, however, should be restricted only to severe thrombocytopenia concomitant with fibrinolysis and microangiopathic anemia as arising in 2 very specific tumors in young children—kaposiform hemangioendothelioma (KHE) and tufted angioma (TE)—pathophysiologically underpinned by DIC precipitated by platelet and clotting factor sequestration [12]. There has yet been no systematic characterization of coagulation abnormalities in angiosarcomas, a phenomenon with undoubted therapeutic and potentially prognostic significance for this tumor. We were thus keen to better define the incidence and clinical course of DIC amongst patients with this rare but often fatal disease, with concomitant evaluation for consumptive coagulopathy in epithelioid hemangioendotheliomas as a clinical comparator group.

## 2. Methods

Consent was obtained from the Institutional Review Board of the Mount Sinai School of Medicine and Hospital (MSSM) for retrospective analysis. All cases with the diagnosis of “angiosarcoma” seen at MSSM between 1 January 2000 and 31 June 2013 were identified through the institutional diagnostic records system. Clinical data were retrieved from patients' clinical notes and the MSSM electronic patient medical records. The cutoff date for reporting data was 31 June 2013. Survival data was determined from medical charts and cross referenced against the death registry.

Overt DIC was defined according to the International Society of Thrombosis and Hemostasis (ISTH) guidelines, evaluating 4 parameters (platelet counts, fibrinogen degradation products, prothrombin time, and fibrinogen levels) and assigning points to particular values (Table 1) [13]. This metric was chosen amongst the several clinical criteria available to define DIC in view of its simplicity and widespread use. Patients with pertinent laboratory derangements were then evaluated for the presence of other causes of coagulopathy or DIC including trauma, sepsis, pharmacotherapeutic myelosuppression, and bone marrow involvement with disease. Patients in whom these causes had been excluded, or in whom these causes existed but were unable to fully account for the hematological abnormalities seen, then had further elements of their clinical course characterized. Patients were designated as having bulky disease if the largest single or contiguous tumor mass measured 5 cm or more in diameter. We evaluated this clinical characteristic as we postulated that the size of singular or contiguous masses (as distinct from the sum total size of multiple disseminated tumor masses) in vascular tumors may influence the likelihood of consumptive coagulopathy putatively because smaller such masses are less likely to trap platelets in sufficient quantity to cause significant platelet trapping and destruction. The 5 cm cutoff was chosen based upon a previous evaluation of KHE in which all patients developing KMP had primary tumors larger than 5 cm [14]. The same process of case identification and evaluation for DIC was then repeated for patients with the diagnosis “epithelioid hemangioendothelioma” (EHE).

## 3. Results

Forty-two patients with angiosarcoma were identified from our institutional database. The median age was 62 years (range 16–86 years); 22 patients (52%) were male. Twenty patients (48%) had advanced disease, defined as either metastatic or unresectable AS. Eighteen patients (43%) had bulky disease, of whom 12 had advanced disease. The five commonest anatomical primary sites include breast (12 patients), liver (5), mediastinum (4), upper limb (3), and scalp or face (3). Twelve patients (29%) had radiation-associated AS, the majority of which developed following radiation for early breast cancer (6 developing breast AS and 2 developing chest wall AS).

**Figure 1:**
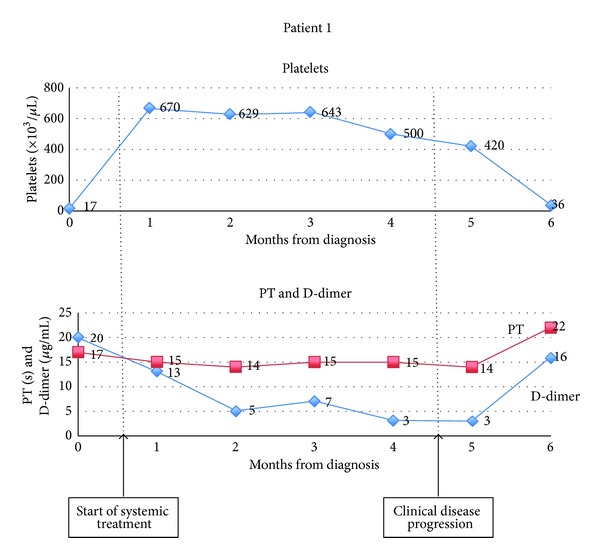
Variation in hematological and coagulation parameters showing coagulopathy at diagnosis that improved with therapy but recurred at disease relapse.

Out of this cohort of AS patients, 11 patients were identified to have evidence of DIC during the course of their illness. Four of these patients were deemed to have had their coagulopathy probably attributable to pharmacotherapy (including chemotherapy) or sepsis, leaving 7 patients (17%) with unexplained DIC. The clinical features of these patients are described in Table 2. The trend of selected hematological and coagulation parameters for 2 representative patients is detailed in Figure 1.

**Figure 2:**
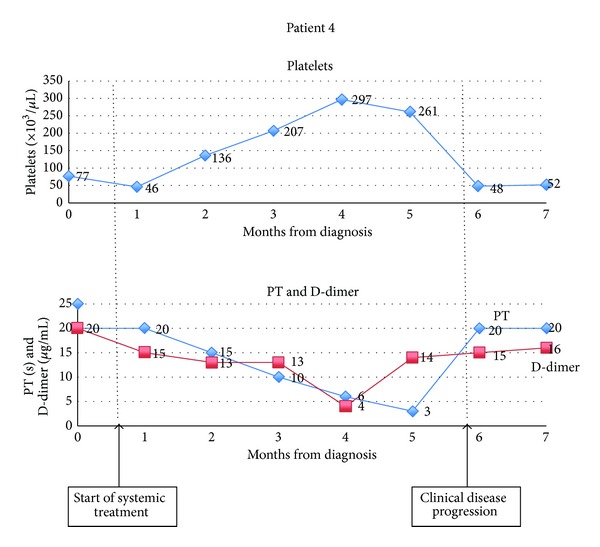
Variation in hematological and coagulation parameters showing coagulopathy at diagnosis that improved with therapy but recurred at disease relapse.

Six patients fulfilled the ISTH criteria for overt DIC; patient 7, whose clinical data was incomplete, scored the maximum 4 points based on the hematologic and coagulation data available. Three patients manifested either clinical thrombosis or hemorrhage during the course of their illness; patients 2 and 4 manifested clinically apparent hemorrhage necessitating local therapy (surgical ablation or radiotherapy); and patient 3 had superior vena caval thrombosis. All but one patient manifested coagulopathy within the first month of diagnosis; all patients who demonstrated clinicoradiologic response or stability to systemic therapy (patients 1, 3, 4 and 5) also demonstrated improvement in their coagulation and hematological parameters in tandem with their clinical response. All patients demonstrated worsening coagulopathy coincident with disease progression. The depth and trend of this coagulopathy for two representative patients is depicted in Figure 2. Only two patients (patients 3 and 4) had bulky disease. All patients had very short survival, with limited duration of disease control, if any.

We conducted an identical review of patients with EHE. There were 17 patients in total. The median age was 51 years (range 23–80 years), and 9 patients (53%) were female. Fourteen patients (82%) had disease arising in the liver, of whom 8 had metastatic disease (primarily to lungs and bone) and 6 had multifocal liver disease. Of the remaining 3 patients (18%) with extrahepatic primary disease, 2 had advanced EHE. Only 3 patients (18%) had bulky disease according to our definition. Patients were variably treated with a range of sequential therapies including orthotopic liver transplantation, hepatic ablative therapies, systemic therapies, or expectant management. Half of the patients were free of disease progression for more than 2 years, and 4 patients were stable for more than 5 years. No patient demonstrated evidence of DIC.

## 4. Discussion

Amongst the malignant mesenchymal tumors, angiosarcomas have a particularly aggressive clinical course. Even in the context of early disease receiving aggressive multimodality therapy, relapses are frequent and often early; in the setting of advanced disease, treatment, responses are uncommon and usually short-lived [5, 15, 16]. Systemic therapy in advanced disease often involves the use of anthracyclines and taxanes, putatively the most active agents in this disease [17]. The value of therapy directed against the vascular endothelial growth factor (VEGF), often overexpressed in this endothelium-derived tumor, remains unsettled. A recent study reported 2 out of 23 angiosarcoma patients achieving a partial response to single agent bevacizumab [18]; these therapies continue to be evaluated actively in this disease. What is not in doubt is the well-documented propensity of anti-VEGF therapies to cause vascular toxicities. Bevacizumab has been associated with bleeding in up to 40% of patients, with the rate for such events with the tyrosine kinase inhibitor sunitinib reported to be 16%; the risk of both arterial and venous thromboembolism can also be increased with use of these agents [19]. The potential for significant myelotoxicity with cytotoxic therapy, as well as the risk of hemorrhage and hemostatic derangements with anti-VEGF therapies, in this aggressive malignancy with few effective therapies, brings into sharp relief the significant therapeutic implications of any disease-associated coagulopathy.

Few systematic data exist on the prevalence of DIC in sarcomas; as mentioned earlier, one series found DIC in 2 out of 41 (4%) sarcoma patients evaluated [3]. More generally, with the possible exception of nonislet cell tumor hypoglycemia seen in a proportion of sarcoma subtypes such as solitary fibrous tumor [20], sarcomas are not known to be consistently associated with particular paraneoplastic phenomena. Whether this is due to a dearth of systematic evaluation in an uncommon and biologically diverse group of diseases, or rather the consequence of a genuine lack of association between sarcomas and the triggering factors of paraneoplastic syndromes, is unknown at this time. Specific to angiosarcoma, the occurrence of consumptive coagulopathy has been documented in several case reports. Two reports described bulky de novo breast angiosarcomas [8, 11], two others reported nonbulky scalp angiosarcomas in elderly Asian males [9, 10], and one case described a bulky hepatic angiosarcoma [7]. The two patients with breast disease and one with scalp disease had concomitant hemorrhage or thrombosis. In the only report that described delivery of antineoplastic therapy, chemoradiotherapy resulted in resolution of DIC for the other patient with scalp disease [9]. In all other cases, patients experienced rapid clinical deterioration and eventual demise.

Seventeen percent of patients in our series developed DIC, with all patients with complete data meeting the ISTH criteria for overt DIC. DIC affected patients across a range of anatomic primary sites, manifested early in the disease, improved with effective therapy, and recurred at the time of disease progression. Importantly, these derangements were assessed to be independent of concomitant clinical conditions known to cause similar laboratory abnormalities, such as sepsis or bone marrow failure. Half the patients developed clinically apparent thrombosis or hemorrhage; given the clear presence of a consumptive coagulopathic process in all patients, it is possible that an occult clot or bleeding diathesis was present in the remaining patients that was merely not assessed for or detected clinically. The clinical course of these patients was not inconsistent with the known dismal prognosis of patients with visceral angiosarcoma, with no patients living beyond 1 year [21].

We did not find any evidence of this coagulopathy in the EHE patients. Notwithstanding clear distinctions in clinical behavior described earlier, EHE is arguably the closest clinical analogue of angiosarcoma. Both are malignant vascular neoplasms of endothelial origin that commonly overexpress similar mitogens like VEGF and have been studied together in therapeutic trials [18]. While the sample sizes in both groups were too small to allow for statistically meaningful comparisons, the disparity in the occurrence of coagulopathy between angiosarcomas and EHEs may point towards underlying biological features unique to the former, as distinct from a feature common to vascular neoplasms.

It is interesting to speculate on the particular features of specific subtypes of mesenchymal tumors that lend them more prone to DIC than others. In the case of KMP, it is speculated that the unique vessel architecture of Kaposiform hemangioendothelioma (KHE) and tufted angiomas (TA) promotes platelet trapping and the resultant consumptive coagulopathy; in contrast to the ordered tree-like vasculature of infantile hemangioma, convoluted capillaries arise directly off large vessels in KHE and TA resulting in turbulent flow-promoting platelet activation and aggregation [12]. The observation of KMP in young infants may suggest the additional role of developmental differences in infantile endothelial structure and composition that increase the likelihood of coagulopathy [12]. Not dissimilarly in angiosarcomas, it is possible that as yet undefined derangements in the physiology and function of the endothelium, the histologic cell of origin in this disease contributes to the platelet adhesion and activation that leads to consequent DIC.

In our series, 71% of patients (5 out of 7) developing DIC had angiosarcoma where no single or contiguous mass was larger than 5 cm. Notably, all patients who developed DIC had metastatic disease. The necessarily tentative nature of any conclusions drawn from this small sample of patients notwithstanding, these data may be interpreted to suggest the limited importance of local physical factors (specifically size of single largest tumor mass) as compared with systemic factors, such as hormonal and cytokine elements associated with disseminated disease, in the development of DIC in angiosarcoma. Furthermore, the apparent correlation with disease activity, in as much as the manner the DIC waxed and waned in tandem with clinical disease, suggests concordance between the processes driving the angiosarcoma with those causing the coagulopathy and hints at the potential utility of the latter as a biomarker for the former. Indeed, though not evaluated in the context of DIC, D-dimer, the final degradation product of cross-linked fibrin that is one of the markers of activated clotting and fibrinolysis, has been shown to have independent prognostic value in sarcomas [22], as in several other solid tumors.

We suggest that these cases represent an addition to the literature linking angiosarcoma to derangements in hemostatic control. Undoubtedly, the severity and clinical manifestations of the coagulopathy vary widely even within this small group, and their prognostic implications remain to be rigorously defined. The precise mechanisms underlying the occurrence of this phenomenon also remain to be elucidated. Nevertheless, we think that this data importantly highlights the presence of a clinical syndrome with clear potential implications for therapy. Angiosarcoma patients are often subjected to aggressive oncologic therapy, often including extensive surgery and intensive chemotherapy; vigilance for the development of DIC and hemorrhage appears critical to minimize the morbidities of these therapies. As consistent with the treatment of DIC in cancer in general, therapy involves addressing the underlying malignant disease with appropriate antineoplastic therapy. In addition, the intriguing hints at potential alterations in tandem with disease burden speak to the possibility of utilizing these parameters as convenient biomarkers of disease status.

## 5. Conclusions

Our data systematically complements previous reports linking angiosarcoma to consumptive coagulopathy. This association appears unique to this histologic subtype amongst the vascular tumors and has clear implications for the safe and effective delivery of therapy. We suggest that a baseline coagulation screen be performed for newly diagnosed patients with angiosarcoma; if abnormal, these tests should be repeated regularly to assess the trend with disease and therapy. Further studies should assess this association in larger data sets with a view to evaluating its true prognostic value, if any.

## Figures and Tables

**Table 1:** International Society of Thrombosis and Hemostasis criteria for overt DIC.

Laboratory test	Result	Score*
Platelet count (×10^3^/L)	>100	0
	>50 but <100	1
	<50	2

Increase in fibrinogen degradation products (FDP)	None	0
	Moderate^@^	2
	Strong^@^	3

Prolongation of prothrombin time (PT) over upper limit normal (s)	<3	0
	>3 but <5.9	1
	>6	2

Fibrinogen level (g/dL)	≥1	0
	<1	1

*Scores ≥5 compatible with overt DIC.

^@^A level of <0.5 *μ*g/mL (the upper limit of normal of the D-dimer assay in this study) was considered normal, a level between 0.5 and 5.0 *μ*g/mL was considered moderately increased, and a level of >5.0 *μ*g/mL was considered strongly increased.

**Table 2:** Hematologic and coagulation parameters associated with clinical features of the 7 angiosarcoma patients with DIC.

No.	Age (yrs), sex (M/F), anatomic sites	Systemic therapy in metastatic disease	ISTH score	DIC in 1st 30 days^*∧*^	DIC resolved with disease response/ stability	DIC recurred at progression	Bone marrow involvement	Tumor burden^@^	Clinical thrombosis/ hemorrhage	Survival in metastatic disease	Remarks
1	37 M liver with metastasis to spleen	Pegylated liposomal doxorubicin	7	Yes	Yes	Yes	No	Nonbulky	No	9 months	Metastatic at first presentation. No evidence of clinically significant portal hypertension

2	42 F breast (de novo) with metastasis to chest wall, ribs, lung	Pegylated liposomal doxorubicin plus carboplatin, sirolimus, pazopanib, abraxane, bevacizumab plus vinorelbine	5	No	NA	Yes	Not done	Nonbulky	Yes	11 months	Localized to right breast at first presentation, had mastectomy, then 6 cycles adjuvant gemcitabine plus docetaxel followed by radiation. Developed metastatic relapse within 11 months

3	39 M mediastinal with metastasis to bone, lung, brain	Doxorubicin plus paclitaxel, ifosfamide plus cisplatin, gemcitabine plus vinorelbine	5	Yes	Yes	Yes	Not done	Bulky	Yes	5 months	Metastatic at first presentation

4	54 F cardiac with metastasis to lung, pleura, bones, bone marrow	Doxorubicin plus ifosfamide	7	Yes	Yes	Yes	Yes	Bulky	Yes	6 months	Metastatic at first presentation

5	70 F breast (radiation related) with metastasis to lymph nodes, lung	Doxorubicin plus ifosfamide	6	Yes	Yes	Yes	No	Nonbulky	No	11 months	Metastatic at first presentation; developed angiosarcoma 9 years following lumpectomy and radiation for early stage breast adenocarcinoma

6	66 F skull and meninges with metastasis to spinal cord, lungs, bone	Nil	6	Yes	NA	NA	Not done	Nonbulky	No	1 month	Metastatic at first presentation; poor performance status precluded any therapy

7	78 M foot (bony) with metastasis to lungs	Nil	4*	Yes	NA	NA	Not done	Nonbulky	No	2 months	Metastatic at first presentation; poor performance status precluded any therapy

*Data available only for prothrombin time and platelets, giving a maximum possible ISTH score of 4.

^@^Bulky tumor is defined as the largest single or contiguous tumor mass 5 cm or more in diameter.

^*∧*^Development of DIC within 30 days of presentation with metastatic disease.
